# Effects of Blue Light Toward Emmetropization

**DOI:** 10.7759/cureus.87714

**Published:** 2025-07-11

**Authors:** Dennis Ee See Ong, Safinaz Mohd Khialdin

**Affiliations:** 1 Department of Ophthalmology, UKM (Universiti Kebangsaan Malaysia) Medical Centre, Kuala Lumpur, MYS

**Keywords:** blue light, emmetropization, hyperopia, illumination, myopia, refractive errors

## Abstract

Emmetropization was thought to result from a genetically determined process until later discoveries found that myopia could be induced, which implied that environmental factors, even modest changes, can affect eye growth under local retinal control. Humans are born with refractive errors and emmetropize into a refractive state of no error during the developmental period. For many years, it has not yet been clearly known how the eye determines the sign of defocus during the developmental period. This review aimed to study the effects of blue light on emmetropization, especially in this digital era where children are being exposed to electronic devices that emit blue light at a very young age. In view of the possible detrimental effects of blue light on the retina, the effects of blue light need to be understood and clearly studied to achieve a balance for the healthy development of the human eyes. The mechanisms of emmetropization and development of refractive errors were studied, and articles involving a wide range of animal models, such as chicks, guinea pigs, monkeys, and tree shrews, were reviewed in this review. Numerous studies collectively revealed that emmetropization is a complicated process involving the interaction of multiple factors, such as longitudinal chromatic aberration (LCA), temporal frequency, bandwidth of light wavelength, light intensity, temporal contrast, circadian rhythm disruption, and hormones. This review focused mainly on chromatic aberration. LCA causes wavelength defocus, leading to refractive changes. It has been observed that chicks, Cichlid fish, and guinea pigs became more hyperopic when exposed to short-wavelength blue light compared to those exposed to red light. The results seemed to be conflicting in tree shrews and rhesus monkeys, but the reasons are still unclear. Experiments have also shown that LCA is not essential for emmetropization, as the eyes of many species could compensate for lens-induced myopia or form deprivation in monochromatic illumination. The measurement of ocular biometry can be influenced by the lighting conditions. For instance, under red or white light, the axial length of the eye tends to increase without any refractive changes.

Conversely, when exposed to blue and white light, the eye length decreases in the presence of positive lens defocus, but this effect is not observed under red light. Furthermore, the choroidal thickness increases when a positive lens is used in red and white light, but no changes occur under blue light. It is important to note that variations in the response of ocular biometry to lighting conditions have been observed among different species. Despite these alterations, the impact of lighting conditions on emmetropization, or the process of achieving normal vision, is temporary and short-term.

## Introduction and background

Emmetropization refers to an active developmental process that utilizes visual cues to align the optical power of the eye with its axial length, ensuring that the unaccommodated eye is focused correctly for distant objects [1, 2]. Newborns often have refractive errors such as myopia (where the eye is typically too elongated for its optical power) or hyperopia (where the eye is too short for its optical power), but most newborns are generally hypermetropic, which is 8-16 times more prevalent, than myopic [3-5]. However, during the developmental period, if the processes that underlie emmetropization are successful, these refractive errors gradually adjust, resulting in the attainment of a state of refractive accuracy with no errors, known as emmetropia [6].

The mechanism by which the eye determines whether the refractive error is myopic or hyperopic remains a fundamental question in the field of emmetropization. Before the mid-1800s, it was widely believed that the regulation of eye size during emmetropization was solely non-visual and predetermined by genetic factors, limited only by the anatomical constraints of the eye socket. However in recent years, it has been reported that spending time outdoors can prevent the onset of myopia and slow the myopic shift in refractive error [7], which subsequently challenged the belief that emmetropization was predetermined by genetic factors, and it was concluded that myopia was the result of eye strain caused by close-up tasks.

Later, studies demonstrated that myopia could be induced in various animal models. Different animal models offer different advantages and disadvantages due to the difference in species: Monkeys have the most similar pathological features to humans, but chicks, rabbits, and mouse models are frequently used in studies assessing optimal performance and cellular or molecular mechanisms of myopia, with more affordable costs compared to monkeys [8]. Although animals are not perfect models of humans, these investigations provide valuable insights into the processes and characteristics of emmetropization, as well as the development of refractive errors, and provide compelling evidence for the significant environmental impact on eye growth [2,8,9]. Furthermore, they lay the groundwork for the development of optical and pharmacological interventions that can effectively reduce the prevalence and progression of myopia, which has become a significant public health concern.

Another significant breakthrough in this field was the discovery that these changes in eye growth could be confined to the specific area of the visual field that lacked spatial vision, even in cases where the optic nerve was sectioned. These findings led to the realization that even minor alterations in the visual environment can impact eye growth, and the retina regulates these changes locally [9]. However, despite years of research, a definitive answer to this question has yet to be fully elucidated. One possible explanation is that the eye relies on changes in the retinal image caused by defocus, in combination with chromatic aberrations, to discern the nature of the refractive error [1, 6].

Reviews from studies seem to support that exposure to white light, such as natural sunlight (without blue light-reducing filters) or artificial lighting that contains a significant amount of blue light, may be crucial for the healthy development of a child’s eye. With the rising concerns on the significant increase in myopia, from 24% to 36%, among children and adolescents between 1990 and 2023 [10] and the increasing exposure to screens and devices which emit blue light, this paper aims to review the findings from studies to investigate how blue light affects emmetropization, in terms of the mechanism involved and how the interactions between blue light and other factors influence emmetropization, with particular emphasis on blue light-induced longitudinal chromatic aberration (LCA), the different wavelengths of light, and temporal frequency.

## Review

Studies published from the year 2000 with related keywords, such as blue light, wavelength, emmetropization, LCA, myopia, and refractive error, were searched using Google Scholar and PubMed. Only studies that discuss the effects of blue light or illumination conditions were included in this review. From the studies being reviewed, factors that affect emmetropization are mainly LCA [9, 11-26], temporal frequency [1, 6, 14, 15, 17, 18, 27, 28], bandwidth (of light wavelength) [14, 19, 27, 29, 30], light intensity [14, 27, 29, 31-33], temporal contrast [14], circadian rhythm disruption, and hormones [34, 35].

Longitudinal chromatic aberration

LCA is the phenomenon where different wavelengths of light cannot be focused onto the same focal plane because shorter wavelengths (such as blue light) are refracted more than longer wavelengths (such as red light). This results in wavelength defocus, which means the blue light focuses closer to the lens and the red light further back [9, 36]. In hyperopic defocus (image behind the retina), red components of the image appear more blurred than blue; the opposite has been observed in myopic defocus [36]. Many studies suggest LCA provides a directional signal that the eye can use to guide emmetropization, adjusting growth to reduce defocus [9, 36, 37].

Refractive changes have been observed in different animal studies in response to the wavelength defocus arising from LCA (Table 1). Chick eyes exposed to blue light showed reduced axial elongation and became more hyperopic, whereas those exposed to red light showed increased axial length and were more myopic [12, 13, 20, 22, 29]. Similar trends were reported in Cichlid fish [21], guinea pigs [23-26], and rhesus monkeys [16]. However, some experiments have shown conflicting results. For instance, Smith et al. [19] found that rhesus monkeys wearing lenses with long-wavelength (red) filters became more hyperopic after 120 days, compared to those wearing lenses without any filters. In another study, Gawne et al. [17, 18] found that tree shrews exposed to red light developed hyperopia, while blue light caused myopia - opposite to the expected LCA effect.

**Table 1:** Responses of different species toward the different factors involved in emmetropization

Factors	Animal Species	Responses
Longitudinal chromatic aberration	Chicks	Show classic LCA response—hyperopia in blue light, myopia in red light [12, 13, 20, 22, 29].
	Cichlid fish	Follow similar trends as chicks, confirming sensitivity to LCA [21].
	Guinea pigs	Also exhibit hyperopia in blue and myopia in red light, consistent with LCA-driven emmetropization [23-26].
	Rhesus monkeys	Mixed results—some studies show typical LCA responses [16], while others (Smith et al.) report unexpected hyperopia under long-wavelength-filtered conditions [19].
	Tree shrews	Contradict expected LCA effects, becoming hyperopic in red light and myopic in blue light [17, 18].
Wavelength of light	Chicks	Show reduced eye growth in blue light, altered by lens defocus and illumination type [10]. Lens compensation occurred in red and white light (positive/negative) but absent in blue (positive) light [12].
	Cichlid fish	Also exhibit reduced eye growth in blue light [21].
	Guinea pigs	Show slower growth in blue light [38]; lens compensation is impaired in both red (positive/negative) and blue (negative) light; choroidal thickening seen under blue light [25].
	Rhesus monkeys	Red light induces greater myopia over time; blue light prevents myopia; differences attributed to L-cone sensitivity [16].
	Tree shrews	Transition from hyperopia to myopia under extended blue light, possibly losing ability to detect defocus sign [11].
Temporal frequency	Chicks	Flickering light and the presence/absence of specific wavelengths (e.g., blue) significantly influenced eye growth and refractive outcomes [15, 27].

Despite these findings, lens-induced myopia and form-deprivation myopia can still be compensated for under monochromatic illumination, suggesting that while LCA can guide emmetropization, it is not necessary for the process [16, 36] in various species. While many animals respond to wavelength defocus by adjusting eye growth in line with LCA predictions, inconsistencies - particularly in tree shrews and some monkey studies - suggest that additional mechanisms are involved. Furthermore, the ability of eyes to compensate for lens-induced defocus even under monochromatic conditions suggests the presence of alternative visual cues beyond LCA in regulating emmetropization.

Effects of blue light versus red light

Generally, blue light causes a hyperopic shift in eyes in monochromatic illuminated conditions compared to red light [12, 13]. Eye growth slows in short-wavelength (blue) light, which affects emmetropization. These effects suggest a role of wavelength in guiding ocular development, possibly via cone photoreceptor stimulation or circadian rhythm modulation [37]. The idea that LCA might influence eye growth has been debated, but it may not be essential for certain ocular responses such as lens compensation or choroidal changes. It is further proposed that different cone pathways might govern changes in choroidal thickness and ocular elongation separately, contributing to wavelength-specific effects [12].

Lin et al. [37] found that axial length grew more in red or white light than in blue light during short-term exposure, with no significant changes in refraction, possibly due to corneal changes [27]. These axial differences diminished over prolonged exposure, suggesting an early effect plateau, after observing in chicks that alterations in refraction and axial growth become less pronounced and noticeable after Day 10 [37]. In experiments applying lens-induced defocus, Rucker and Wallman [12] observed that in blue light, chick eyes showed decreased axial length with positive lenses but not with negative lenses. Under a red light, no significant axial change occurred regardless of defocus.

Choroidal responses also varied: In blue light, thickness did not differ between positive and negative lenses; in red and white light, it thickened with positive and thinned with negative lenses. Lens compensation occurred in red and white light, regardless of defocus sign, but was absent in blue light with positive lenses [12]. In guinea pigs, Jiang et al. [25] found impaired lens compensation in red light (both lens types) and impaired compensation in blue light for negative lenses. They also observed choroidal thickening under blue light, in contrast with Rucker and Wallman’s findings.

In rhesus monkeys, red light induced greater myopic shift over time compared to white and blue light. By week 22, monkeys reared in blue light did not develop myopia, while two red-light-reared monkeys exhibited significant myopia, possibly due to enhanced L-cone sensitivity [16]. Another finding indicated no difference in mean refraction between blue and white light groups, suggesting that emmetropization might be insensitive to chromatic focus differences [16].

Gawne et al. [11] observed tree shrews shifting from hyperopia to myopia under prolonged blue light exposure, implying an eventual inability to determine defocus sign under sustained monochromatic conditions. This suggests a critical period during which monochromatic light affects emmetropization, with diminished effects beyond that period, possibly due to circadian influences [11, 37].

These findings suggest that short-wavelength light (blue) generally inhibits axial eye growth across species, contributing to a hyperopic shift. This is likely mediated by cone-specific pathways and possibly circadian rhythms. Species differences in cone distribution and sensitivity (e.g., L-cone dominance in monkeys) may explain variable responses to red light. The observed inconsistencies in lens compensation and choroidal response (e.g., between chicks and guinea pigs) indicate that emmetropization mechanisms are multifactorial and species-specific. Furthermore, the presence of a critical period suggests that the timing of monochromatic exposure is crucial for its effects on eye development. The lack of consistent changes under red light and the absence of lens compensation in some blue light conditions suggest that LCA is not the sole driver of emmetropization.

Influence of temporal frequency

Emmetropization is a complex, multifactorial process that includes not only the influence of spatial visual cues but also temporal ones. Temporal frequency, which describes how quickly light intensity changes over time (such as flickering light), has been shown to interact with the spectral content of illumination to affect eye growth. Rucker et al. proposed that the effects of wavelength (color of light) and flicker frequency combine in modulating ocular development, particularly eye length and refraction [15, 27].

In controlled experiments, Rucker et al. exposed White Leghorn chicks (Gallus gallus domesticus) to various temporal frequencies of light (0, 0.2, 1, 2, 5, and 10 Hz), with 0 Hz representing steady (non-flickering) light and 10 Hz representing rapidly flickering light. The results showed that under short-wavelength light (blue/yellow), there were no significant differences in eye length between 0 and 10 Hz (269 ± 16 μm vs. 224 ± 12 μm, P < 0.01). However, under long-wavelength light (red/green), it was observed that significantly greater change in eye length occurred under steady light (336 ± 31 μm) compared to flickering light (218 ± 20 μm, P < 0.01) [15].

In a follow-up study, it was reported that refraction did not change across temporal frequencies when blue light was present. However, in the absence of blue light, there was a hyperopic shift (>+1D) at high frequencies and a myopic shift (<-0.6D) at low frequencies [27].

These findings reinforce that emmetropization is influenced by both spectral (color) and temporal (flicker rate) properties of light. In particular, blue light may serve a protective role in preventing excessive eye elongation and myopic shifts under constant lighting conditions. Furthermore, flickering light appears to reduce eye growth more effectively under red/green light than under blue/yellow light, suggesting that long-wavelength-induced eye growth is more sensitive to temporal modulation. These interactions highlight the complex and dynamic nature of visual processing in eye development and suggest that both wavelength and temporal frequency must be considered in efforts to control refractive error development.

Table 2 summarizes the results of studies on the effect of chromatic aberration on emmetropization in various species. Different ocular components, such as axial or eye length, choroidal thickness, vitreous length, anterior and vitreous chamber depths, were regulated by these light exposures. Results showed that blue (short-wavelength) light generally slows eye growth and prevents myopic progression.

**Table 2:** The effects of chromatic aberration on emmetropization in different species from various studies

Author (Year)	Title	Study Subjects	Results
Seidemann and Schaeffel (2002) [13]	Effects of longitudinal chromatic aberration on accommodation and emmetropization	Chicks	Raised chicks in red light and found that they develop about one diopter more myopia than those raised in blue light.
Rucker and Wallman (2008) [12]	Cone signals for spectacle-lens compensation: differential responses to short and long wavelengths	Chicks	Compensation for lens-induced defocus in blue light primarily occurred through changes in eye length. Compensation for lens-induced defocus in red light mainly involved changes in choroidal thickness. Lens compensation in white light involved changes in eye length and choroidal thickness. The fact that lens compensation was more effective in white light compared to monochromatic illumination suggests that longitudinal chromatic aberration may play a role in lens compensation. There was no significant difference in refractive compensation between white light and monochromatic red light, regardless of whether positive or negative defocus was present. However, compensation occurred in blue light with negative but not positive defocus.
Long et al. (2009) [23]	Illumination with monochromatic long-wavelength light promotes myopic shift and ocular elongation in newborn pigmented guinea pigs	Guinea pigs	Monochromatic long-wavelength light illumination developed significant myopia with increased anterior chamber depth, lens thickness, and vitreous chamber depth compared with those raised in mixed-light illumination. Supports the hypothesis that monochromatic long-wavelength light may affect the normal process of emmetropization and cause a myopic shift.
Qian et al. (2013) [38]	Transfer from blue light or green light to white light partially reverses changes in ocular refraction and anatomy of developing guinea pigs	Guinea pigs	The eyes of animals reared under broadband white light underwent a gradual developmental decrease in ocular refraction (emmetropization) accompanied by an increase in the vitreous length. Relative to animals reared under broadband white light, animals reared under monochromic middle-wavelength light (530 nm) became more myopic, and those in monochromic short-wavelength light (430 nm) became more hyperopic.
Foulds et al. (2013) [20]	Progressive myopia or hyperopia can be induced in chicks and reversed by manipulation of the chromaticity of ambient light	Chicks	Red light induced progressive myopia. Progressive hyperopia was induced by blue light.
Jiang et al. (2014) [25]	Interactions of chromatic and lens-induced defocus during visual control of eye growth in guinea pigs (Cavia porcellus)	Guinea pigs	Exposure to red light induced more myopic refractions over time, deeper vitreous chambers, and increased axial lengths, compared to blue and white light exposure. Blue light inhibited negative-lens-induced axial myopia and slowed axial eye growth. Plus lenses could no longer induce hyperopia under red light.
Liu et al. (2014) [24]	The effects of monochromatic illumination on early eye development in rhesus monkeys	Rhesus monkeys	No significant difference in mean refraction between the blue and white light groups. Emmetropization is insensitive to the chromatic difference in focus between blue and white light.
Rucker et al. (2015) [27]	Blue light protects against temporal frequency-sensitive refractive changes	Chicks	The absence of blue light leads to changes in refraction that are sensitive to temporal frequency. Blue light prevents temporal frequency-sensitive changes in eye growth. Blue light induces a decrease in anterior chamber depth, typically resulting in a decrease in the eye's power and a hyperopic shift, especially at higher temporal frequencies. During emmetropization, the presence of blue light reduces corneal astigmatism. The presence of blue light prevents both hyperopic and myopic shifts.
Rucker et al. (2018) [14]	The role of temporal contrast and blue light in emmetropization	Chicks	In white light, the shorter focal plane of blue light serves as a signal that slows down eye growth. Exposure to low temporal frequencies enhances eye growth, but this effect is observed specifically in yellow light, not white light. White light offers color contrast, luminance contrast, and light vergence cues that contribute to accurately achieving emmetropization and preventing myopia development.
Rucker et al. (2018) [15]	Color and temporal frequency-sensitive eye growth in chicks	Chicks	Exposure to high temporal frequencies reduced eye growth, while low temporal frequencies increased eye growth. The average increase in eye length was lower when high temporal frequency stimuli were applied. The average change in eye length was lower in the blue/yellow condition compared to the red/green condition. The presence of the blue component in the light source contributed to a reduction in eye growth. At low temporal frequencies, the smallest increase in eye length was observed with blue light, while the greatest increase occurred when there was no blue light component. The choroid tends to thicken more at intermediate frequencies with blue light. In contrast, without blue light, the choroid thins, leading to an increase in the depth of the vitreous chamber in elongated eyes. The myopic defocus caused by blue light, resulting from longitudinal chromatic aberration, leads to a decrease in eye growth.
Wang et al. (2018) [22]	Effects of light of different spectral composition on refractive development and retinal dopamine in chicks	Chicks	Chicks that are raised under blue and ultraviolet (UV) lighting conditions exhibit a lower occurrence of deprivation myopia compared to those raised under red and white light. Additionally, eyes with normal vision tend to become more hyperopic (farsighted) after being exposed to blue and UV lighting.
Wu et al. (2018) [39]	Myopia prevention and outdoor light intensity in a school-based cluster randomized trial	School children	School children with longer outdoor time in school (200 minutes) showed significantly less myopic shift and axial elongation compared with the control group and a 54% lower risk of rapid myopia progression.
Zou et al. (2018) [26]	Effect of altered retinal cones/opsins on refractive development under monochromatic lights in guinea pigs	Guinea pigs	Guinea pigs developed relative hyperopia in the short-wavelength group and relative myopia in the middle-wavelength group. The axial length of the short-wavelength group was shorter relative to that of the white light group. Corneal curvature, anterior chamber depth, and lens thickness showed no significant differences among the three groups.
Tian et al. (2019) [28]	Wavelength defocus and temporal sensitivity affect refractive development in guinea pigs.	Guinea pigs	The refraction in blue light was more hyperopic than in green light at all temporal frequencies. Weakening of the wavelength defocus signal means that the refractions in monochromatic blue light became less hyperopic, and the refractions in monochromatic green light became less myopic than in steady light.
Lin et al. (2020) [37]	Effect of duration and temporal modulation of monochromatic light on emmetropization in chicks	Chicks	Chicks raised under blue light demonstrated less axial growth than those raised under red and white light. Refractions were more hyperopic (farsighted) in the presence of blue light compared to red light. There was no significant difference in choroidal thickness between red and blue light conditions. Prolonged exposure to monochromatic light, especially red light, increased eye growth. After a short exposure, there was no difference in axial length between blue and red light. However, after more prolonged exposure, axial length changed more in red light compared to blue light. Choroidal thickness increased more in the presence of red light compared to blue light. Both steady and flickering light conditions led to a greater increase in axial length and vitreous chamber depth in chicks exposed to red or white light compared to those exposed to blue light.
Lou et al. (2020) [40]	Effects of narrowband light on choroidal thickness and the pupil	Adult humans	Following exposure to broadband light, red light, and dark conditions, the choroid experienced a significant thinning compared to its state before exposure. However, no substantial change in choroidal thickness was observed after blue light exposure. Short-term exposure to long and short wavelengths (red and blue light, respectively) elicited different effects on choroidal thickness. Exposure to narrowband blue and red light resulted in distinct changes in choroidal thickness.

## Conclusions

To conclude, LCA plays a significant yet non-essential role in guiding emmetropization across various animal species. Studies consistently demonstrate that short-wavelength blue light generally induces hyperopia and reduced axial growth, while long-wavelength red light promotes myopia and increases eye elongation. However, variations exist depending on species, duration of exposure, and other factors such as temporal frequency, light intensity, and circadian influences. While LCA can guide refractive development, compensation mechanisms persist under monochromatic illumination, suggesting that emmetropization relies on multiple, interacting pathways. Further research is needed to fully understand the interplay of chromatic and non-chromatic cues.

As we are currently emerging into the world of the digitalized era, exposure to electronic devices emitting blue light is almost inevitable for anyone, even at a younger age. Although prolonged exposure to blue light is known to cause retinal damage (which has not been discussed in this review), the role of blue light in achieving emmetropization and the maintenance of emmetropia cannot be undervalued. While the underlying mechanisms and principles of emmetropization between animals and humans are similar, the findings from animal studies need to be carefully translated and applied to humans; ideally, more human studies are also needed to validate the findings.
